# A Retrospective Cross-Sectional Population-Based Study on Prenatal Levels of Adherence to the Mediterranean Diet: Maternal Profile and Effects on the Newborn

**DOI:** 10.3390/ijerph15071530

**Published:** 2018-07-19

**Authors:** Isabel Peraita-Costa, Agustín Llopis-González, Alfredo Perales-Marín, Ferran Sanz, Agustín Llopis-Morales, María Morales-Suárez-Varela

**Affiliations:** 1 Unit of Public Health and Environmental Care, Department of Preventive Medicine, University of Valencia, Avinguda Vicente Andrés Estellés s/n, Burjassot, 46100 Valencia, Spain; ivperaitacosta@hotmail.es (I.P.-C.); agustin.llopis@uv.es (A.L.-G.); agustinllopis@gmail.com (A.L.-M.); 2 CIBER in Epidemiology and Public Health (CIBERESP), Av. Monforte de Lemos, 3–5, Pabellón 11, Planta 0, 28029 Madrid, Spain; 3 Department of Obstetrics, La Fe University Hospital, Avinguda de Fernando Abril Martorell, 106, 46026 València, Spain; perales_alf@gva.es (A.P.-M.); ferran.sanz@hotmail.com (F.S.)

**Keywords:** Mediterranean diet, small for gestational age, pregnancy, primary prevention

## Abstract

The Mediterranean diet (MD) is a dietary pattern with important benefits. The objectives of this study were to assess the adherence to the MD among pregnant women in Valencia (Spain) and characterize the pregnant women according to their level of adherence. Finally, we aimed to examine the role of MD adherence during pregnancy in the anthropometric development of the newborn. The study included 492 pregnant women who were followed at La Fe Hospital in 2017. The self-administered “Kidmed” questionnaire for data collection on dietary information evaluation was used and a clinical history review of mothers and newborns was performed. Two groups of mothers were identified: those with low adherence (LA) and optimal adherence (OA). The study revealed that 40.2% of the women showed LA to the MD. The newborns born to these women presented a higher risk of being small for gestational age (SGA) {adjusted odds ratio (aOR) = 1.68; 95% confidence interval (CI) 1.02–5.46} when adjusting for parental body mass index (BMI) and multiple gestation, but not when adjusting for all significant possible confounders (aOR = 2.32; 95% CI 0.69–7.78). The association between MD and SGA was not significantly affected by the use of iron and folic acid supplements (aOR = 2.65; 95% CI 0.66–10.65). The profile of the pregnant woman with LA is that of a young smoker, with a low level of education and a low daily intake of dairy products. These results suggest that LA to the MD is not associated with a higher risk of giving birth to a SGA newborn.

## 1. Introduction

A healthy diet is an important part of a healthy lifestyle at any time, but is especially vital when pregnant or planning a pregnancy. Eating healthily during pregnancy will help the baby to develop and grow correctly. Fetal growth problems may be suggestive of environmental exposure, which may affect the physiology of organ systems, leading to fetal growth retardation and increased risk of chronic diseases in later life [1,2].

Maternal nutrition has been recognized as one of the most important extraneous stimuli influencing fetal growth and development and it is considered one of the most important modifiable factors during pregnancy, a time period in which a greater contribution of nutrients and energy is required to meet the demands of fetal development [3,4]. Thus, good maternal nutritional status, together with other healthy life factors, helps maintain a correct balance in fetal nutrition and endocrine status during pregnancy, which is essential to the health of the mother and child [5]. Traditionally, dietary assessment in pregnant women was performed by analyzing the intake of certain foods and energy content together with the contribution of micro- and macronutrients [6,7]. By not considering the maternal diet as a whole, possible interactions between foods and nutrients could be overlooked [8], and hence there is a growing interest in the study of dietary patterns in pregnancy [3,7,9].

The Mediterranean diet (MD) is a dietary pattern reflecting the different customs and cultural interrelations of the civilizations that have developed in the Mediterranean basin over the years. Traditionally, it has been characterized as a diet based on high intake of vegetable foods (oil, fruits, vegetables, legumes, cereals, nuts), moderate intake of dairy products, fish, poultry, and eggs, and low intake of red meats [10]. Given this, the MD seems to guarantee a caloric and nutrient supply in sufficient quantities and adequate proportions [11]. This dietary pattern is low in saturated fatty acids, rich in carbohydrates, fiber, and antioxidants, and has a high content of monounsaturated fatty acids and n-3 polyunsaturated fatty acids, which are primarily derived from olive oil and fish intake [12].

The effects of adherence to the Mediterranean pattern during the perigestational period have been studied. An association with lower risk of preterm birth [5,9,13,14], miscarriage [15,16,17], hypertensive disorders [18], or gestational diabetes [19,20], as well as lower weight gain in these women [5,21,22] has been suggested. Likewise, it has also been associated with a lower risk of congenital malformations such as spina bifida [23] or cardiac defects [24], as well as a lower risk of intrauterine growth restriction [9,21,25,26,27] and even long-term effects such as better bone quality [28,29], and lower risk of development of atopy [30,31] and/or abdominal obesity in childhood [32]. To the best of our knowledge, there is no prospective data on the impact of maternal adherence to an MD during pregnancy on anthropometric measures in their offspring.

However, all these benefits are lost in today’s society, in which this traditional diet pattern is increasingly less common. In Spain in particular there has been a remarkable evolution in the patterns of food intake in the last 40 years, in all age ranges [33,34]. Healthy life habits are being abandoned, increasingly approaching a westernized pattern, with high intake of refined sugars and foods of animal origin, especially red meat and derivatives, and a decrease in intake of foods of plant origin, leading to an increased presence of saturated fats and cholesterol in the diet [33,35,36].

This trend seems to affect even pregnant women and it would therefore be appropriate to identify the profile of women at greater risk of low adherence (LA) versus optimal adherence (OA) and to raise awareness about the value of good nutrition and its impact of the development of the newborn [3,37].

Low birth weight (LBW) is usually defined as birth weight of a live born infant below 2500 g [38]. LBW infants define a heterogeneous group: some born too early, some born at term but small for gestational age (SGA), and some both born too early and SGA [39]. SGA newborns are those who are smaller in size than normal for gestational age, most commonly defined as a weight below the 10th percentile for gestational age. However, the designation has prognostic importance because it predicts susceptibility to hypoglycemia, hypothermia, and polycythemia [40]. LBW and SGA are recognized as a disadvantage due to risk of early growth retardation, fast catch-up growth, infectious disease, developmental delay, and death during infancy and childhood, as well as development of obesity and non-communicable diseases later in life [41]. On the other hand, and due to the disparity of results in the available literature on this topic, it is also important to continue studying the effects of the MD and other nutritional factors during pregnancy on the health of the newborn.

Given all the information currently available, the hypothesis of this study is that LA to MD could negatively impact the development of the newborn and that less educated and younger women would be the most likely to maintain a LA to MD during pregnancy. The objectives of this study were to assess the adherence to the MD among pregnant women in Valencia (Spain) as well as to characterize the pregnant women according to their level of adherence. Finally, we aimed to examine the role of MD adherence during pregnancy on the anthropometric development of the newborn.

## 2. Materials and Methods

### 2.1. Study Design

The study here presented is a retrospective cross-sectional population-based study examining maternal dietary habits during pregnancy (MD) and evaluating the effects on the newborn.

In the descriptive phase, the characteristics of the parents, clinical and obstetric history of the mother, and data related to gestation and delivery, including the state of the newborn (gestational age, height, weight, cranial perimeter, Apgar scores, gasometric analysis results etc.) were analyzed. In the analytical phase of the study, the information collected previously was assessed in relation to the mothers’ adherence to the MD and the possible effects on the newborn.

Thus, considering adherence to the MD as the variable of exposure, a Kidmed questionnaire of 16 items was used to assess usual dietary intake during pregnancy. Women with scores Kidmex index scores > 7 were assigned to the OA group and those with scores ≤ 7 to the LA group. The information corresponding to these two groups has been obtained before classification, thus ensuring as much as possible the homogeneity of the population sample, and its representativeness of the population as a whole.

### 2.2. Study Population

All mother–child pairings admitted after delivery to the Obstetrics Ward of La Fe Hospital in Valencia and belonging to the health coverage area of this hospital were considered for the study population during a 6-month period in 2017. In total, 579 mothers were subsequently enrolled and 492 provided complete outcome data after signing consent to participate in the study (a participation rate of 85%).

**Figure 1:**
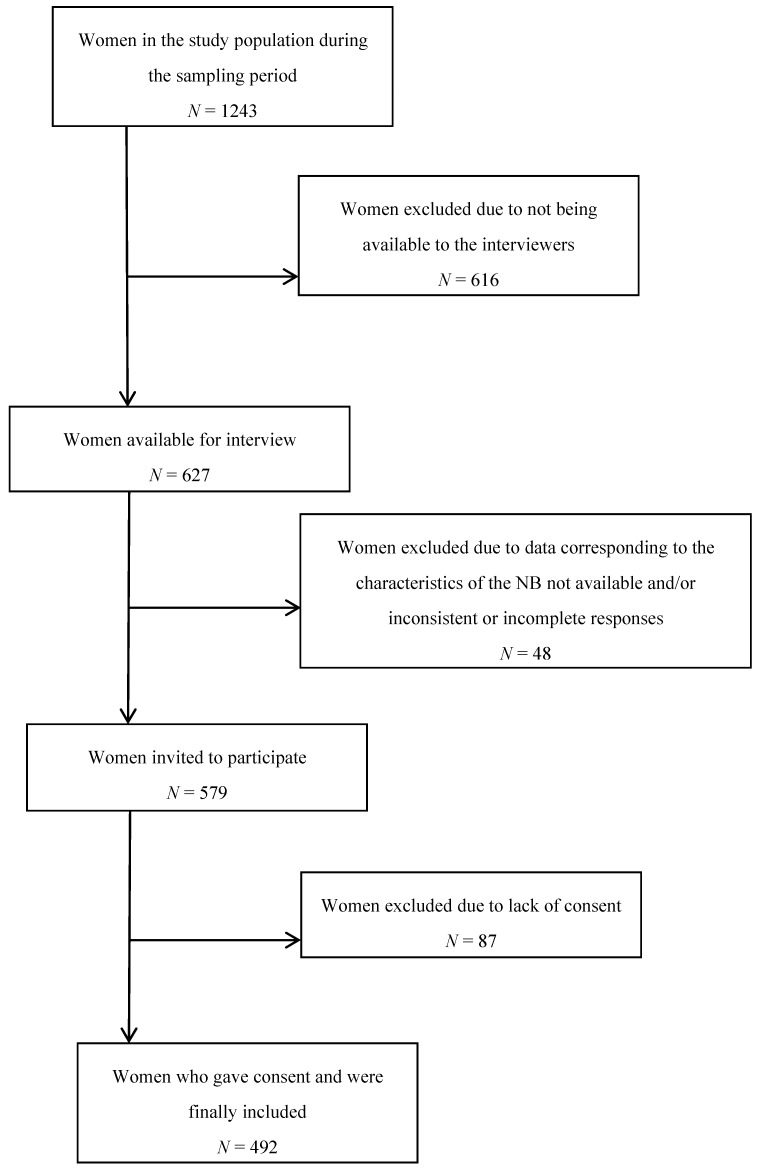
Selection of subjects.

Mother–child pairs were excluded from the study if data corresponding to the characteristics of the newborn was not available to be consulted and collected and also if the mother’s responses were inconsistent or incomplete (eight mothers were excluded) (Figure 1).

All subjects gave their informed consent, which included a confidentiality agreement of the data collected according to Organic Law 15/1999 of December 13 of Protection of Data of Official Nature, for inclusion before they participated in the study. The study was conducted in accordance with the Declaration of Helsinki, and the protocol was approved by the Ethics Committee of La Fe Hospital (CEIC 2014/0116).

### 2.3. Collected Data

The technique employed to collect the information used in this study was a direct and personal interview with the mothers after birth and the subsequent review of the corresponding clinical records for both mothers and newborns. Interviewers were medical students trained by dieticians on as to how conduct the interviews. Information on mother’s age, country of origin, education, marital status, employment status during the pregnancy, maternal physical activity, maternal diseases, parity, mothers’ cigarette exposure, drug use, alcohol use, coffee intake, caffeine drinks, prenatal vitamin use, dairy products at breakfast, and daily dairy intake was obtained through questionnaires administered after birth. The questionnaires were revised by the dieticians that trained the medical students. From the clinical history, weight and height measurements for the mother and father were available, and the body mass index (BMI) was calculated for both. Obstetric and neonatal data was also obtained from the clinical history.

#### 2.3.1. Nutrition Assessment

For each participating mother, the usual daily intake during the pregnancy was measured with a shorter Spanish version of the Kidmed index [42]. This test was developed to quickly and easily assess the degree of adherence to the MD [42]. The Kidmed test has been successfully tested in numerous studies [42,43,44,45,46,47,48]. The degree of the mother´s adherence to a traditional MD was assessed based on the intake of dietary compounds positively associated with the MD. As such, intakes of vegetables, legumes, fruits, nuts, cereal, fish, dairy products, and oil were assigned a value of +1, whereas intakes of compounds with a negative association (sweets, fast foods) were assigned a value of −1. Serra-Majem et al. [42] splits adherence into three levels: ≥8 optimal; 4–7 improvement needed, and ≤3 poor. In this study, the sample was split into two levels: >7 optimal and ≤7 low. The OA group included all those that would have been considered optimal by Serra-Majem and the LA group all those that would have been included in the improvement needed and poor group by Serra-Majem. The division into two groups instead of three was done in part to avoid problems due to the sample size that would complicate analysis, and the division was deemed appropriate as a way to identify those women that might benefit from nutritional intervention.

#### 2.3.2. Parental Data

Sociodemographic, lifestyle, dietary habit, and anthropometric data of both parents was also collected during the interviews with the mothers. With regard to the anthropometric data collected, BMI was calculated as weight in kilograms/height in m^2^. Pre-pregnancy weight was obtained through medical records. The value taken was that reported in the last record available before pregnancy confirmation and all were within the one year prior to pregnancy. Total maternal pregnancy weight gain was taken from clinical records. Physical activity contemplated four possible levels of activity depending on the total time of physical activity in both leisure and working hours on an average day. As per the scores obtained by the women, four categories were established—none: total exercise score < 0.5; light: total score ≥ 0.5 but <1; moderate: total score ≥ 1 but <2; and intense: total score ≥ 2.

With respect to the clinical and obstetric history of the mother, information was collected on previous pregnancies, differentiating between primigravida and multigravida women. Similarly, for the parity variable we distinguished between primiparous or multiparous women. Another factor that was taken into account was the number of previous miscarriages suffered by the mothers.

#### 2.3.3. Newborn Data

Given the heterogeneity in the literature on the effects of maternal diet on the newborn, information about the newborns was collected to search for a possible association between maternal diet and effects on the newborn. The information was extracted from the interviews with the mothers as well as by reviewing the clinical history of the newborn.

The data collected included anthropometric values of the newborn such as weight, height, and cephalic perimeter while ensuring consideration of the sex of the newborn, since it is a determining factor when analyzing these values. Newborns were classified as SGA when their weight was lower than the 10th percentile for the newborn’s gestational age as compared with that expected for the same sex and gestational age according to the Spanish standards [49]. Data on whether there were problems at birth, as well as whether the newborn was admitted to the Neonatal Ward or the Neonatal Intensive Care Unit, was collected. Apgar score (heart rate, muscle tone, reflexes, and skin color) for the neonate was also recorded [50,51,52]. The data from gasometric analysis in arterial and venous blood of the umbilical cord was also recorded at the time of delivery (including pH, PO_2_, and PCO_2_) since it is a method that objectively evaluates the state of the newborn [53] and is used systematically at the La Fe Hospital in Valencia.

### 2.4. Statistical Analysis

First, the primary exposure variable of interest was adherence to a MD for the mothers during their pregnancy. A descriptive study of the population studied for each level of adherence to MD was carried out (LA vs. OA) and the outcomes of interest were the mother’s sociodemographic information, habits, and obstetric profile. The characteristics of the newborn with special attention to relation with SGA were analyzed. For the comparison of the different variables in the levels of adherence to maternal MD, an ANOVA for the quantitative variables and the *X^2^* test for the qualitative variables were used. Considering the adherence to the MD as the independent variable, an analysis to determine the presence of statistically significant differences in the study variables was performed.

Multivariate logistic regression models were further performed to examine the association between mother’s adherence to MD and the outcome of interest (SGA) after adjusting for confounders. We created six adjusted models; the following variables were considered as potential confounding factors: parental BMI, multiple gestation, iron supplementation intake, and folic acid supplementation. Odds ratios (OR), adjusted odds ratio (aOR), and 95% confidence intervals (95% CI) were computed to estimate the degree of association. All hypothesis testing was conducted assuming a 0.05 significance level and a two sided alternative hypothesis.

Finally, to determine the relationship between adherence to the MD and the risk of giving birth to a SGA newborn, crude odds ratios (ORs) were calculated using binary logistic regression models. To account for the effects of several potential confounders simultaneously a conditional multiple logistic regression model was used to obtain the adjusted odds ratio (ORa), using different models in relation with the characteristics identified as significantly different between the LA and OA groups. The confidence interval (CI) applied in both cases was 95%. The attributable risk (AR) of SGA which was considered attributable to LA to MD was also calculated. Attributable risk was calculated using the following formula: $\frac{RR-1}{RR}*100=AR\%,$ with RR referring to relative risk [54]. In studies in which the outcome (SGA) occurs in less than 10% of the unexposed population (4.2% in this study) the OR provides a reasonable approximation of the relative risk. 

All analyzes were performed using the IBM SPSS Statistics 22 software (SPSS Inc., Chicago, IL, USA), considering as significance level *p* < 0.05.

## 3. Results

The sociodemographic and lifestyle characteristics during the pregnancies of mothers who participated in the study are shown in Table 1. In the present study, the Kidmex index scores ranged from −2 to 12, and were categorized into two groups: >7, OA to MD and ≤7, LA to MD. Table 1 presents the degree of adherence to the MD of the 492 pregnant women included in this study; 40.2 (*n* = 198)% met criteria of LA and 59.7% (*n* = 294) that of OA. The overall mean score was 6.80 ± 2.28, with the OA group having a mean score of 8.90 ± 0.85 and the LA group having a mean score of 5.23 ± 1.68 (*p* ˂ 0.001). In the LA group, the profile participants were significantly younger, with the most notable difference appearing in women ≤24 years old, who accounted for 17.2% of pregnant women with LA versus only 6.1% of pregnant women with OA. There were no significant differences in marital status or nationality. However, 86.4% of pregnant women with LA versus 80.3% of pregnant women with OA were Spanish. Participants with LA were significantly less educated; 28.8% of LA had no or primary studies versus 12.3% in the OA group. Pregnant women with LA were significantly less employed, less physically active, and more likely to smoke before and during the pregnancy. Maternal drug use appeared only in the LA group (*n* = 2) and no differences were observed in alcohol use during pregnancy.

Table 2 shows the maternal dietary habits according to their adherence to the MD. No significant differences were observed between LA and OA groups for the intake of coffee, tea, or chocolate. Caffeinated drinks were consumed more by the LA group. Significant differences were found in folic acid supplement use and iron supplement use, with a lower intake observed in the LA group. No significant differences appeared in the use of prenatal vitamins. The intake of fish was lower in the LA group. In the LA group, 17.7% did not consume a dairy product at breakfast, versus 8.8% of the OA. The LA group also presents a significantly lower intake of dairy products.

In this study no statistically significant differences were found for the anthropometric values (maternal height, pre-pregnancy maternal weight, pre-pregnancy maternal BMI, maternal weight at delivery, maternal BMI at delivery, weight gained during pregnancy, paternal height, paternal weight, and paternal BMI) of the parents according to the level of adherence. However, if we group overweight (BMI: 25.00–29.99) and obese (BMI ≥ 30.00) groups together we find statistically significant difference, with a higher incidence of BMI ≥ 25 in the LA group. In both groups, the weight gain during pregnancy was adequate in most women according to Institute of Medicine recommendations [55], although it was slightly higher in the OA group (60%) versus the LA group (59%). In the OA group, 18% of women had weight gain above the recommended values and 22% had weight gain below the recommended values, while in the LA group, 22% of women had weight gain above the recommended values and 19% had weight gain below the recommended values.

There were no statistically significant differences in the maternal obstetric factors (length of pregnancy, previous gestations, parity, previous miscarriages, in-vitro fertilization (IVF), multiple gestation, and hospitalization during pregnancy) or the characteristics of the newborn (sex, weight, height, cranial perimeter, SGA, birth complications, newborn admitted to neonatal ward, Apgar scores, gasometric analysis) between the LA and OA groups. Even though it is not statistically significant, it must be noted that the percentage of newborns admitted to the neonatal ward was more than double for those born to mothers with LA than for those born to mothers with OA.

We investigated the possible different effects of LA to MD in newborns delivered pre-term and at term. As seen in Table 3, the risk of having SGA newborns in mothers with LA to MD is only significant when adjusting for parental BMI and multigravidity (aOR = 1.68 CI 1.02–5.46). If adjusted also for all the possible confounders identified previously as significantly different among the two groups (mother’s age, education, employment, physical activity, smoking, and caffeinated drinks), there is no association between LA and SGA (aOR = 2.32; 95% CI 0.69–7.78). Once this value was obtained, it was also adjusted for the use of iron and folic acid supplements to see if this had any effect on the association; the use of either supplement individually or their combination resulted in no significant change (aOR = 2.65; 95% CI 0.66–10.65).

Attributable risk quantifies the proportion of SGA which is attributable to a LA to MD, which in this study was 40.48%. In other words, the attributable risk is the proportion by which the risk of SGA can be reduced if the adherence to the MD is that of the reference category, which would be OA in this study.

## 4. Discussion

In the present study, we observed that 40.2% of pregnant women had an LA to the MD during pregnancy. Given that this is a population-based study, we can theorize that the prevalence of LA to MD among the Valencian population studied is of around 40%. Studies that report on the diet quality of the Spanish population as a whole using the Kidmed index have not been found.

However, there are studies that assess the adherence to MD that may be used to know if the LA to the MD of this study is comparable that of the general adult population in Spain. In one study, MD was assessed with the MD Adherence Screener (MEDAS) [56] score; in another, 54% [33] of a representative sample of the Spanish adult population was found to not reach modest MD adherence. Another study found that non-adherence to the MD among the population of the Balearic Islands was 56.9% [57]. Another study carried out in the Balearic Islands showed that Balearic islanders had 63% non-adherence to MD [58].

In addition, there are some studies that report adherence to MD in certain sectors of the Spanish population. One study reported on the adherence to MD of nursing students in one region of Spain and showed that 56.6% of the sample would fall into our LA category [43]. In another study carried out in healthy children of a region of northern Spain, 53.3% of the sample would be considered LA according to our criteria [59]. Compared with these studies, pregnant women seem to have greater adherence to MD than other sectors of society.

Studies that use the same index to classify adherence to MD of Spanish or European pregnant women have not been found. There are studies that use other indices of classification of adherence to the MD to report on the level of adherence in pregnant women (from Guipuzcoa, Asturias, Valencia, Catalonia (Spain) and Crete in Greece) [25] with results (~44% LA) similar to those obtained in our study. Another study showed that 36.1% [60] of the mothers in Menorca (Spain) had a low quality Mediterranean diet during pregnancy according to the Mediterranean Diet Score [61]. Another study found that 43.23% of women from southern Spain did not adhere to the MD [62].

In this study and when adjusting for parental BMI and multigravidity, women with LA have a greater risk of having SGA newborns; however, the saturated adjusted model finds no association between LA and SGA. This lack of an association between LA and SGA found in this study is nuanced and therefore must be interpreted carefully. The use of a saturated adjustment model for a sample size like the one of this study may introduce errors in the results. Dietary patterns are usually accompanied by other factors that may be an important source of confounders [3,21,33,35,63], making the isolation of the effects of one single factor difficult and more so due to possible synergistic or antagonistic relations among the factors. It would be advisable to hesitate in stating that there is no association between LA to MD and SGA newborns given the issue of the sample size and evidence from previous studies to the contrary.

Other studies have found that adherence to MD protects against SGA [64]. In a review study, the dietary patterns associated with lower risk of having SGA babies were named differently, but had similar characteristics across studies. These were most importantly high intakes of fruits, vegetables, and dairy foods [41]. Dietary patterns labeled “Mediterranean” were positively associated with higher birth weights [41]. In an intervention study where both the intervention group and control group were given the same basic MD recommendations but the intervention group was also advised to consume at least 40 mL of extra virgin olive oil and a handful (25–30 g) of pistachios daily, there was a significant decrease in SGA in the intervention group [65]. One study showed that the relative risk of giving birth to a SGA baby was significantly lower in women who consumed >60 g/d of seafood than in women who consumed ≤5 g/d (OR = 0·56 (95% CI 0·35, 0·88) [27]. A Spanish study on seafood consumption and birth weight found that higher maternal intakes of crustaceans and canned tuna, but not other types of seafood, were associated with increased risk of SGA [66]. Finally, there is also a study that found that the relative risk of SGA, birthweight, and infant growth were not associated with maternal diet [67].

Several studies have demonstrated that the high nutritional quality of the MD is devoid of risks of inadequate intake for several vitamins and minerals [68,69]. It is well established that during gestation, essential nutrients are transferred from the maternal to the fetal circulation across the placenta [70].

The finding of younger women with lower levels of education showing less adherence to the MD ratifies the results of previous studies [3,7,21,33,63,71,72]. One possible explanation is that this profile would correspond to women less aware of the importance of diet for their health and that of their newborn. It also seems to reflect the problem of the westernization of the lifestyle, which is increasingly distant from the traditional pattern [33,35].

Educational level and employment status can be indicators of the socioeconomic level, which has been associated with adherence to a given dietary pattern [7,63,73]. A significant difference was found in this study for employment status, and since higher quality products tend to be the most expensive, it seems logical that women of lower socioeconomic level would resort to cheaper and lower quality products [73].

Different studies associate LA to a healthy diet with unhealthy practices such as smoking and physical inactivity [3,21,33,35,63]. In the present study there were significant differences, with the LA group presenting a lower level of physical activity and higher proportion of smokers, corroborating previous results [3,21,33,35] and reaffirming the importance of identifying this profile of pregnant women at risk.

Pregnancy is a period in which changes in certain dietary habits [74], such as intake of caffeine, are recommended [75,76,77,78,79]. The WHO recommends an intake lower than 300 mg/day during pregnancy [80], which seemed to have been respected by a majority of the women in this sample without significant differences. This which supports the results of previous studies [21,74]. There is a significant difference in the intake of cola drinks. It seems important to control this growing trend for cola, not only for the amount of caffeine but also for the rest of its components, such as high sugar content [81].

Micronutrient deficiencies are increasingly common, especially in women of childbearing age [5,82], and may be exacerbated during gestation by increased nutritional requirements [83,84]. Some studies have reported a greater contribution of some of these micronutrients (vitamins C, E, B, folate, magnesium, calcium, iron, vitamin D or zinc) associated with the MD [11,13,71,85].

The Ministry of Health, Social Services, and Equality (MSSSI) recommends taking a supplement of 0.4 mg of folic acid daily for at least one to two months prior to conception and to maintain its intake at least until the end of the first trimester; however, the current trend is to maintain supplementation throughout pregnancy [86]. In this study, it was found that among women with LA to the MD, the frequency of folic acid supplementation was lower, a trend already seen in the study by Timmermans et al. Women potentially at risk for folic acid deficiency due to an inadequate diet normally do not compensate this by taking supplements [13,73,87]. For folic acid, factors such as pregnancy planning or maternal characteristics [88] may come into play, so that in those in which supplementation is not started periconceptionally, it may not be started at all, therefore explaining the lower frequency of use among pregnant women in the LA group of the present study.

For iron, due to conflicting results [89], the MSSSI suggests not routinely supplementing pregnant women [86] and reserving supplementation for those women with iron deficiency during gestation. However, in this study, the women potentially at risk for iron deficiency due to LA to MD [11,90] had a significantly lower rate of use of iron supplements.

In a recent review, Haider et al. found possible benefits of using multivitamins including folic acid and iron. This could have had positive effects on mothers with LA and their newborn, thus compensating for possible dietary deficiencies. The finding that women at risk for folic acid and iron deficiency due to their inadequate diet are significantly less likely to take supplementation suggests that an increase in use of dietary supplements within the LA group could positively affect mothers and newborns. In this study, folic acid and/or iron supplementation did not significantly affect the risk of having SGA newborns for women in the LA group. This may suggest that a poor dietary pattern cannot be fully compensated by the use of single nutrient supplements. This statement would be reasonable taking into account that any given dietary pattern encompasses the intake of a large and diverse group of nutrients, all with specific functions.

It is also interesting to look at this issue from another perspective—the protective effect of OA to the MD. The protective effect of OA is about ORa = 0.74 and moves to ORa = 1.0 with the addition of folic acid or iron supplementation. If iron or folic acid supplementation were to be put in a model alone without MD, they could be significantly protective. This could mean that the micro minerals and not the diet affect the decrease in SGA. Therefore, it might be said that LA to the MD may be associated with a greater risk of SGA newborns due to a poor content in essential micronutrients and not due to the dietary pattern as a whole. This hypothesis would need to be tested in future studies centered on supplementation independent of diet.

In this study, both groups have a significant majority of women within the recommendations of total weight gain during pregnancy and there are no significant differences in the number of women who meet the recommended weight gain among the two groups [55]. In the OA group, more women have weight gain below the recommendations than above, while the opposite occurs in the LA group, with more women above the adequate weight gain range than below it. This suggest that women in the LA may be consuming higher calorie diets which lack other essential nutrients. Several studies have shown that adequate weight gain in pregnancy could reduce obstetric and neonatal complications [91,92,93]. This seems to be consistent with previous results [5,21], thus reaffirming the potential protective role of the Mediterranean diet. The low energy content, low glycemic load, high water content, and the high prevalence of fiber-rich vegetable products that characterize this dietary pattern seem to be responsible for this protective role [22].

No significant differences in the anthropometric parental data were found, and therefore the comparability among the two groups was very high in this regard. Special focus was placed on paternal BMI, since no previous studies have been found in which this was taken into account. When studying the BMI values for the fathers, the averages fall into the overweight range in both groups, suggesting that poor adherence to the MD is a problem present throughout the family nucleus. This emphasizes the importance of an intervention in this risk group to raise awareness of the benefits of a quality diet.

Other studies [21,94] have found a greater presence of primiparous women in the LA group, while there are also studies that go in the opposite direction, associating greater parity and lower adherence to a healthy pattern [7,95]. In the sample studied, there is no significant difference in adherence in relation to gravidity or parity. However, it seems logical that there would be greater awareness among multiparous women due to information acquired in previous pregnancies. Likewise, the fact of having more children at home could also contribute to greater care being taken with food, favoring a greater adherence to the Mediterranean pattern in this group [96].

There were no significant differences in the duration of pregnancy, which contrasts with the results of other studies in which adherence to the MD was beneficial in preventing preterm delivery [5,13].

As for the characteristics of the newborns, no significant differences were found in the anthropometric measurements of the newborn. This non-association between diet quality and anthropometric measurements of the newborn contrasts with previous studies [3,71,97] suggesting that a healthy and quality diet favors adequate fetal growth during pregnancy.

The lower frequencies of problems at birth and infant hospitalization in the OA group support the benefits of the MD in pregnancy [18,91,92,93], as well as on the newborn in the short and long term [24,28,30,60]. However, once again in this study no difference was found among the two adherence groups.

The mean scores for the Apgar test in the first and fifth minutes were adequate in the sample studied. In this sense, there are no studies with consistent results that relate the Apgar test and the maternal diet during gestation [98]. The Apgar score cannot be interpreted in isolation for the diagnosis of perinatal asphyxia [52,99]. For this reason, data was also collected on cord blood gasometry, providing an objective measure of the fetal condition prior to birth [53]. In the present study, the means of arterial analysis of both adherence groups were within the mean values, with no significant differences among the adherence groups. No studies have been found with which to compare these results of cord blood gases, perhaps because it is a practice that is normally reserved for cases in which there may be an adverse prognosis for the newborn due to some intrapartum event [53].

Intrapartum fetal asphyxia is an important perinatal complication, and the diagnostic criteria are pH, Apgar score, neurological manifestations (hypotonia, convulsions or coma), and dysfunction of two or more organs [100,101]. Therefore, it may be interesting to take these factors into account when considering new studies.

## 5. Strengths and Weaknesses

One of the strengths of the present study was its performance on a population of pregnant women at the La Fe Hospital in Valencia, following homogeneous selection criteria. Similarly, all the data was collected using the same questionnaire by three identically trained interviewers in a personal and direct way with the mothers, and later contrasted and/or completed with the data of the clinical history record of the mother and the newborn. Another strength is having used the degree of adherence to a nutritional standard for nutritional assessment, rather than for separate foods or nutrient intake, since this approach allows for the evaluation of the diet as a whole, including possible interactions between foods [8,21]. Another relative advantage of this is the greater ease in managing dietary patterns in clinical practice [21]. In addition, variables such as anthropometric measures of the father, the Apgar index, or cord blood gas analysis have been taken into account. This appears not to have been the case in previous similar studies.

Regarding weaknesses, it should be noted that the sample size obtained was not very large, thus limiting the study since appropriate control for confounders is limited, so it would be advisable to increase it to obtain more consistent and reliable results. Likewise, collecting the data after birth could ignore the possible variations during gestation, important especially for the variables related to dietary habits [9]. However, in a longitudinal study by Cuco et al. [102], dietary patterns were studied in different periods of pregnancy without finding significant differences. Data was obtained retrospectively and may be affected by information and/or recall bias.

The Kidmed index used here in pregnant women was validated for a population aged between 2 and 24 years old [42,47]. However, previous studies have shown that the use of a frequency-of-use questionnaire is an appropriate tool to obtain reliable estimates of intake during pregnancy [22,25]. There are also several studies on pregnant women which have used indices for the general population, with adaptation for the exclusion of alcohol use [7,71,94]. However, in the Kidmed test alcohol is not included [42].

## 6. Conclusions

In our population, LA to the MD pattern among pregnant women is around 40%. In conclusion, in the present study LA to a MD was not associated with a higher risk of giving birth to a SGA newborn. However, given contradictory evidence and that the small sample studied here that does not allow for categorical affirmations, LA could still be a risk factor for SGA newborns, and therefore, it would be beneficial to implement early intervention programs that raise awareness among pregnant women of the importance of adopting a healthy lifestyle, adhering to a balanced diet, and compensating possible micronutrient deficiencies. The identification of the profile of the pregnant woman at risk of LA, which is that of a younger woman, with lower level of education, who smokes, is not very physically active, is unemployed, and consumes a low quantity of dairy products, would help identify the target patients for this early intervention. Further studies are needed to better understand the mechanisms of the effect of diet on SGA newborns and the most relevant window of exposure. Further follow-up of this cohort will allow for more accurate determination of the effects of adherence to the MD and if these effects persist in older children.

## Figures and Tables

**Table 1:** Maternal sociodemographic data.

	Low Adherence *N* = 198 (40.2%)		Optimal Adherence *N* = 294 (59.7%)		*p* ^a^
	*n*	%	*n*	%	
Maternal age					
**Years (Mean ± SD)**	31.1 ± 5.6		33.3 ± 4.8		0.001
≤24	34	17.2%	18	6.1%	0.001
25–29	40	20.2%	43	14.6%	
30–34	70	40,5%	103	35.0%	
≥35	54	27.3%	130	44.2%	
**Marital status (Single)**	92	46.5%	120	40.8%	0.126
**Maternal origin**					
Africa	6	3.0%	14	4.8%	0.489
Asia	1	0.5%	1	0.3%	
America	15	7.6%	31	10.5%	
Europe	176	88.9%	248	84.4%	
**Maternal education**					
Without studies	11	5.6%	2	0.7%	0.001
Primary education	46	23.2%	34	11.6%	
Secondary education	97	49%	125	42.5%	
University or postgraduate education	44	22.2%	133	45.3%	
**Maternal physical activity**					
None	70	35.4%	65	22.2%	0.006
Light	97	49.0%	166	56.7%	
Moderate	31	15.2%	62	21.2%	
**Maternal employment status (Employed)**	106	53.8%	206	70.1%	0.001
**Maternal smoking before pregnancy (Yes)**	76	50.7%	63	30.3%	0.198
**Maternal smoking during pregnancy (Yes)**	45	30,6%	30	14.6%	0.001
**Maternal drug use (Yes)**	2	1.5%	0	0%	0.319
**Maternal alcohol use (Yes)**	1	0.8%	2	1.0%	0.640

^a^*p*-value obtained by ANOVA (*p* < 0.05) for the quantitative variables, and by the *X*^2^ test (*p* < 0.05) for the qualitative variables.

**Table 2:** Maternal dietary habits.

	Low Adherence *N* = 198 (40.2%)		Optimal Adherence *N* = 294 (59.7%)		*p* ^a^
	*n*	%	*n*	%	
**Coffee**	68	42.8%	99	42.7%	0.507
**Tea**	12	8.6%	26	12.9%	0.139
**Caffeinated drinks ^b^**	68	43.9%	63	28.9%	0.002
**Hot chocolate**	65	35.5%	105	39.6%	0.307
**Dietary supplement intake**					
Prenatal vitamins	33	25.4%	58	33.5%	0.080
Folic acid ^c^ supplement	36	25.2%	65	34.2%	0.048
Iron ^d^ supplement	100	60.2%	165	70.2%	0.025
**Dairy product at breakfast**	163	82.3%	268	91.2%	0.001
**Daily dairy intake**					
<2 yogurts and/or 40 g cheese per day	85	42.9%	51	17.3%	0.003
≥2 yogurts and/or 40 g cheese per day	113	57.1%	243	82.7%	
**Fish**					
<2 times per week	111	56.1%	87	29.6%	0.001
≥2–3 times per week	87	43.9%	207	70.4%	

^a^*p*-value obtained by ANOVA (*p* < 0.05) for the quantitative variables, and by *X*^2^ test (*p* < 0.05) for the qualitative variables. ^b^ Caffeinated drinks NOT including coffee such as soft drinks/soda/pop/sugary drinks/fizzy drinks and energy drinks. ^c^ (2S)-2-[(4-{[(2-amino-4-hydroxypteridin-6-yl)methyl]amino}phenyl)formamido]pentanedioic acid. ^d^ Ferrous fumarate, ferrous gluconate, ferrous succinate, and ferrous sulfate.

**Table 3:** Risk of small for gestational age infants due to low adherence to Mediterranean diet.

	Low Adherence *N* = 198 (40.2%)			
	Crude OR	95% CI	Adjusted OR	95% CI
Small for Gestational Age				
**No**	1.0 Reference		1.0 Reference	
**Yes**	0.97	0.5–2.31	1.68 ^a^	1.02–5.46
			2.32 ^b^	0.69–7.78
			2.65 ^c^	0.66–10.65

^a^ Adjusted for parental BMI and multiple gestation. ^b^ Adjusted for parental BMI, multiple gestation, mother’s age, education, employment, physical activity, smoking, and caffeinated drinks. ^c^ Adjusted for parental BMI, multiple gestation, mother’s age, education, employment, physical activity, smoking, caffeinated drinks, folic acid, and iron supplementation. OR: odds ratio; BMI: body mass index.
